# Expression and significance of miR - 20b in retinal photoreceptor cells exposed to PCB_1254_

**DOI:** 10.18632/aging.102360

**Published:** 2019-10-16

**Authors:** Xin Zhang, Qingyu Zhang, Yue Jiang, Shuchun Zhang, Qin Hong, Xirong Guo, Xia Chi, Meiling Tong

**Affiliations:** 1 Department of Child Health Care, The Affiliated Obstetrics and Gynecology Hospital of Nanjing Medical University, Nanjing 210004, China; 2 Department of Child Health Care, Nanjing Maternity and Child Health Care Hospital, Nanjing 210004, China; 3 Department of Pediatrics, Nanjing Medical University, Nanjing 210029, China; 4 Department of Child Health Care, Northwest Women’s and Children’s Hospital, Xian 710061, China

**Keywords:** PCB _1254_, miR-20b, MAPK signaling pathway

## Abstract

Previous studies have shown that PCB_1254_ has an adverse effect on zebrafish retinal development, but the basic mechanism behind it is not clear. The purpose of this study was to investigate the molecular mechanisms of PCB-induced retinal dysplasia. RT-qPCR, immunoblotting, HE staining and immunofluorescence were adopted to detect the expression at mRNA and protein level. Functional experiments were carried out in 661w cells including CCK-8 assay, caspase-3 assay, and the flow cytometry, while the functional role of miR - 20b was further investigated by using the zebrafish model. The result showed that PCB_1254_ exposure inhibited cell proliferation and increased the apoptosis of the 661w cells, and the dose–response relationship between the retinal development-related genes (SWS1, CRX, Rho), miR-20b expression and PCB_1254_ exposure was also discovered. We confirmed that miR-20b targeted FGF2 and GRB2 by constructing a dual luciferase reporter gene and suppressed the cell function as well as PCB_1254_. In the miR-20b overexpression zebrafish model, we found abnormal retinal morphology characterized by sparse and irregular photoreceptor cells and the thick photoreceptor cell layers. Our results demonstrate for the first time that PCBs target the MAPK/ERK signaling through miR-20b, affecting retinal cell development and leading to visual impairment.

## INTRODUCTION

Amblyopia is a common non-infectious eye disease in children in China and the incidence of amblyopia is approximately 1.47%–2.8% [1]. If amblyopia is not detected and treated timely, it will cause low vision or even blindness in children, and amblyopia is also closely related to the occurrence of glaucoma and cataract in adults. Although the screening and treatment of amblyopia in children has achieved great progress in recent years, its etiology and pathogenesis remains largely unknown. There are many causes of amblyopia and it is reported that retinal dysplasia or lesions is responsible for the occurrence of amblyopia. Studies have shown that that the age of 0–3 is a critical period for retinal development and visual function in children [2] and children are highly sensitive to toxins and chemical contaminants compared with adults. However, knowledge of environmental factors contributing to eye development is still quite limited.

Polychlorinated biphenyls (PCBs), as common environmental pollutants, are widely found in soil, sediments, and air, as well as in marine and freshwater ecosystems [3]. PCBs have long half-lives, and they are extremely lipophilic, chemically stable, and thermostable, but poorly metabolized, resulting in their bioaccumulation in animal and human tissues [4, 5]. It has been reported that continued exposure to PCBs can lead to impaired visual system development in children [6]. Besides, previous studies have shown that PCB_1254_ exposure can affect the differentiation of retinal layers and adversely damage the photoreceptor cells. In addition, exposure to PCBs can result in abnormal optomotor responses (OMRs) of zebrafish larvae [7]. However, the molecular mechanisms of PCB-induced retinal dysplasia remain unclear.

Many environmental toxicants can interfere with biological functions by regulating microRNA (miRNA) expression [8]. MiRNAs are endogenous, non-coding, single-stranded RNAs that regulate gene expression through translational repression or mRNA degradation [9], and are involved in many physiological, pathological, and biochemical processes such as tumorigenesis, organ development, cell proliferation and differentiation, and apoptosis [10]. MiR-20b, closely associated with environmental toxins, is encoded by the miR-106a-363 cluster and located on the human X chromosome [11, 12]. It has been reported that miR-20b exert important functions in diabetic retinopathy and retinitis pigmentosa [12, 13]. The purpose of this study was to investigate the potential effects of PCB_1254_ exposure in vitro on retinal development and the underlying mechanisms in relation to miR-20b-mediated pathways.

## RESULT

### Toxicity assessment of PCB_1254_ on 661w cell

**Figure 1:**
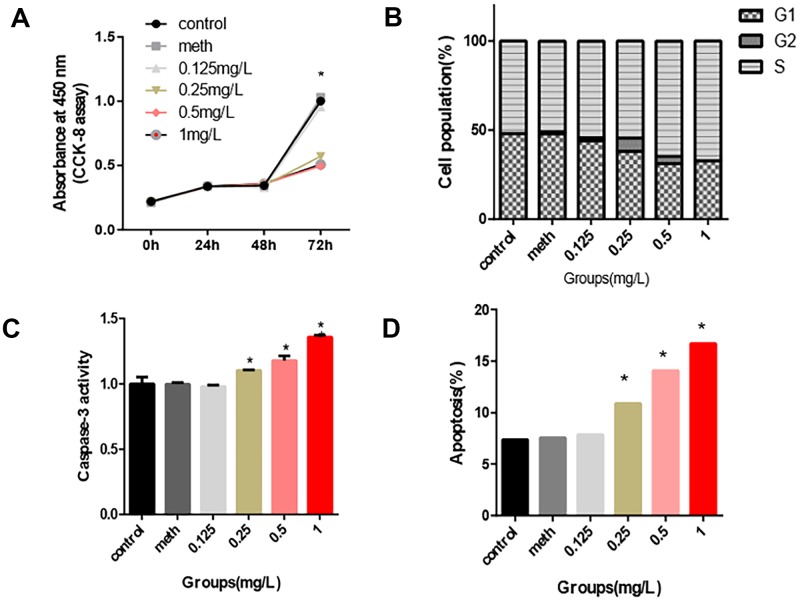
**Inhibition effects of PCB_1254_ on 661W cell.** (**A**) After 72 hours of exposure, the cell proliferation assay showed that as the concentration of PCB_1254_ increased, cell proliferation was significantly inhibited. (**B**) After 72 hours of exposure, cell cycle was not significantly disturbed by PCB_1254._ (**C**–**D**) Apoptosis was measured by flow cytometry and Caspase-3 activity measurement. The results showed that with increasing PCB_1254_ concentration, the number of apoptotic cells increased significantly. (*P<0.05).

**Figure 2:**
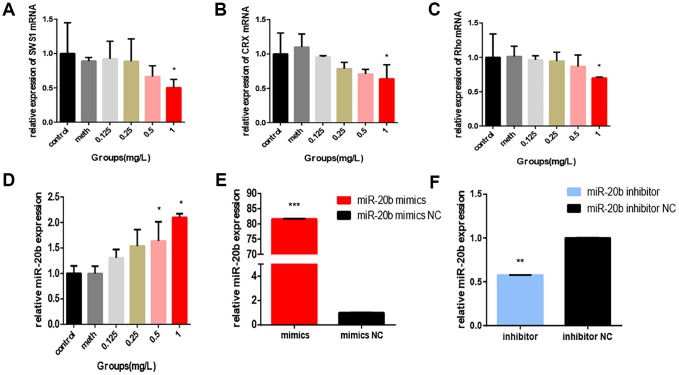
**PCB_1254_ inhibits the expression of related genes.** (**A**–**C**) Total RNA was isolated from 661W cells exposed to different concentrations of PCBs for 24 hours. Photoreceptor cell-specific gene (SWS1, CRX, Rho) expression levels were measured by real-time RT-PCR. The results showed that: As PCB concentration increased, RNA Expression levels gradually decreased (statistically significant in the 1 mg/L group). (**D**) After 24 hours of exposure, total RNA was isolated from the cells and real-time RT-PCR results showed that: expression of miR-20b was increased with increasing concentrations (0.5mg/L group, 1mg/L group was statistically significant), (**E**–**F**) Confirmation of miR-20b overexpression and silencing.

661w cells were used to confirm the toxic effects of PCB_1254_ on retinal photoreceptors. The results showed that continuous exposure to PCB_1254_ significantly inhibited the proliferation of 661w cells. At 72 hours, PCB_1254_ significantly inhibited cell proliferation (Figure 1A). In the apoptosis assays, as the PCB_1254_ concentration increased, the number of apoptotic cells increased, and the caspase-3 activity significantly increased. It was also shown that more and more cells were arrested in S phase as the PCB_1254_ concentrations gradually increased (Figure 1B–1D). It was reported that CRX, SWS1 and Rho are specific markers of retinal photoreceptor cells [14, 15]. It was found that PCB_1254_ decreased the mRNA expressions of CRX, SWS1 and Rho in the 1mg/L concentration group, the other groups have a decreasing trend, but no difference (Figure 2A–2C). These results indicated that PCB_1254_ exposure may affect photoreceptor cell development.

### Expression of miR-20b in cells exposed to PCB_1254_ and its effects on cell function

**Figure 3:**
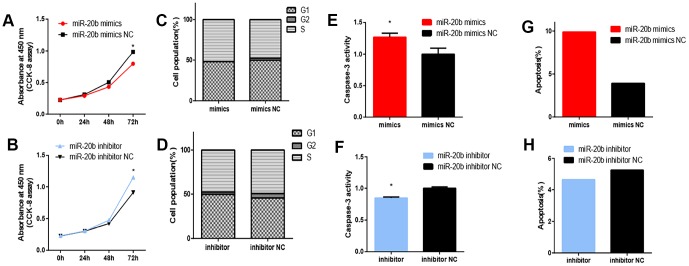
**Effect of miRNA-20b on 661W cell.** (**A**–**B**) After 72 hours of transfection, cell proliferation was inhibited in the miR-20b overexpression group and promoted in the silencing group. (**C**–**D**) After 72 hours transfection, the miR-20b overexpression and silencing groups had no significant change in cell cycle. (**E**–**F**) Apoptosis was detected in two groups of cells by measuring Caspase-3 activity. The results showed that apoptosis was increased in the miR-20b overexpression group, and the silencing group had the opposite effect. (**G**–**H**) Apoptosis was measured in two groups by flow cytometry. The results showed that the proportion of apoptotic cells in the miR-20b overexpression group was significantly increased, and there was no significant difference in the silencing group. (*: P<0.05).

Regarding whether miR-20b levels were affected by PCB_1254_ exposure, RT-qPCR was used to assess the mRNA levels in 661w cells after being exposed to different concentrations of PCB_1254_ for 24 hours. As the concentration increased, we detected an increase in the expression of miR-20b (Figure 2D). To confirm the cellular function of miR-20b, overexpression and silencing plasmids were used to transfect 661w cells (Figure 2E–2F). By using the CCK-8 proliferation assay and cytotoxicity assay, it was found that miR-20b overexpression significantly inhibited cell proliferation, whereas miR-20b silencing had the opposite effect compared with the controls (Figure 3A–3B). The cell cycle assays showed that miR-20b overexpression or silencing did not have a significant effect on the cell cycle (Figure 3C–3D). The PE/7-AAD probe and caspase-3 activity assays were used to test the cell apoptosis, and the results indicated that miR-20b overexpression promoted the cell apoptosis (Figure 3E–3H).

### FGF2 and GRB2 are direct targets of miR-20b

**Figure 4:**
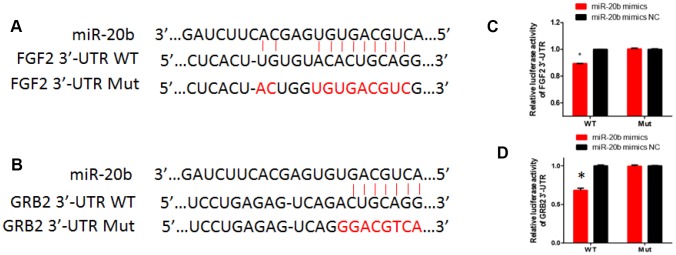
**The regulatory relationship between FGF2, GRB2 and miR-20b.** (**A**–**B**) Diagram of 3’UTR sequences of FGF2 and GRB2 targeted by hsa-miR-20b. (**C**–**D**) HEK293T cells grown to 90% confluency were co-transfected with psiCHECK-2-FGF2 or psiCHECK-2-GRB2 reporter, and the indicated concentration of miR-20b mimics or mimics NC; 24 hours after transfection, luciferase activity was detected using Dual-Luciferase Reporter Assay System according to the manufacturer’s instruction.

FGF2 and GRB2 were identified as potential targets of miR-20b by using TargetScan 5.1 and miRanda. To confirm the target genes of miR-20b, double luciferase reporter plasmids were constructed. The results showed that the luciferase reporter activities for psiCHECK-2-FGF2-3'UTR and psiCHECK-2-GRB2-3'UTR were significantly decreased in the miR-20b overexpression group, whereas in the mut-psiCHECK-2- group, indicating that FGF2 and GRB2 were direct targets of miR-20b (Figure 4).

**Figure 5:**
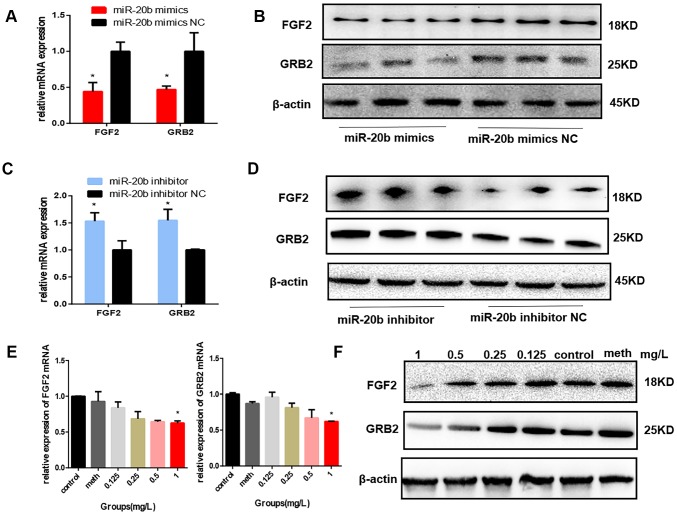
**FGF2 and GRB2 are the target genes of miR-20b.** (**A**–**B**) 24 hours after transfection, the mRNA and protein levels of FGF2 and GRB2 in the miR-20b overexpression group were significantly reduced. (**C**–**D**) The opposite effects were observed in the silencing group. (**E**–**F**) After 24 hours of exposure, FGF2, GRB2 expression levels were decreased with increasing concentrations (statistically significant in the 1 mg/L group). (*: P<0.05).

To determine the effect of miR-20b on FGF2 and GRB2 expressions, mmu-miR-20b mimic, mimic NC, mmu-miR-20b inhibitor, and inhibitor NC were respectively transfected into 661w cells and the mRNA and protein expression levels were detected after 48 h transfection. The results showed that in the miR-20b overexpression group, the mRNA and protein levels of FGF2 and GRB2 were both significantly lower than the control group, while in the miR-20b silencing group, the results were reversed (Figure 5A–5D). What’s more, these results were consistent with the expression levels of FGF2 and GRB2 in the PCB_1254_ exposed group (Figure 5E–5F).

### The effects of FGF2 and GRB2 on cellular functions

**Figure 6:**
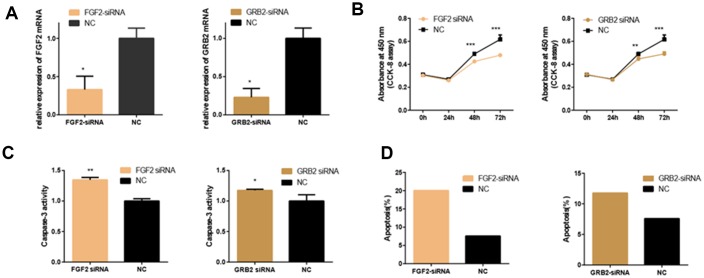
**Effects of FGF2 and GRB2 Silencing on 661W Cell.** (**A**) mRNA expression of FGF2 and GRB2 after siRNA-FGF2 or siRNA-GRB2 and NC transfection. The results showed that mRNA expression was reduced in the interference group. (**B**) In the interference group, cell proliferation was inhibited at both 48 and 72 hours. (**C**–**D**) Apoptosis was increased in the interference group compared with the control group. (*: P<0.05).

The expression of FGF2 and GRB2 were knocked down by using specific siRNAs to deeply explore their effects on 661w cells. First, the interference efficiency was verified by detecting the expression of the corresponding mRNAs (Figure 6A). Then, CCK-8 assays, the PE/7-AAD probe and caspase-3 activity assays were used to detect the changes in cell function. It was showed that knockdown of FGF2 and GRB2 inhibited 661w cell viability and proliferation, and promoted cell apoptosis (Figure 6B–6D).

**Figure 7:**
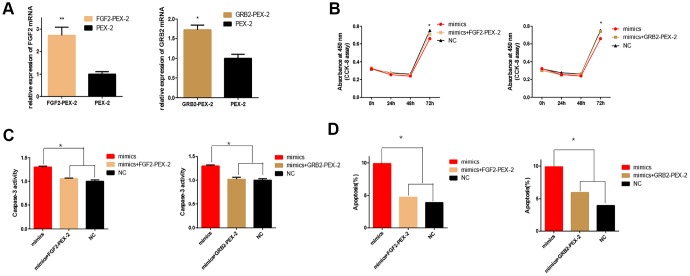
**Overexpression of FGF2 and GRB2 attenuated the adverse effects of miRNA-20b on 661W cells.** (**A**) mRNA expression levels of FGF2 and GRB2 after transfection with PEX-2-FGF2 or PEX-2-GRB2 and PEX-2 as controls. The results showed that the expression of mRNA increased significantly in the overexpression group. (**B**) The miRNA-20b mimics co-transfected with PEX-2-FGF2 or PEX-2-GRB2 significantly increased the proliferation of cells compared to the miRNA-20b mimic group. (**C**–**D**) Groups co-transfected with miRNA-20b mimics and PEX-2-FGF2 or PEX-2-GRB2 groups reduced the effect of the miRNA-20b mimics on apoptosis. (*: P <0.05).

Then, to investigate whether overexpression of FGF2 and GRB2 could rescue the inhibitory effect of miR-20b on 661w cells. The cells were co-transfected with FGF2-pEX-2 and miR-20b mimics, GRB2-pEX-2 and miR-20b mimics to re-express FGF2 and GRB2 in cells, respectively (the overexpression efficiency of FGF2 and GRB2 were examined, Figure 7A). Subsequently, it was found that overexpression of FGF2 and GRB2 partially rescued the anti-proliferative, pro-apoptotic effects of miR-20b in 661w cells (Figure 7B–7D).

### PCB_1254_ regulated MAPK/ERK through miR-20b.

**Figure 8:**
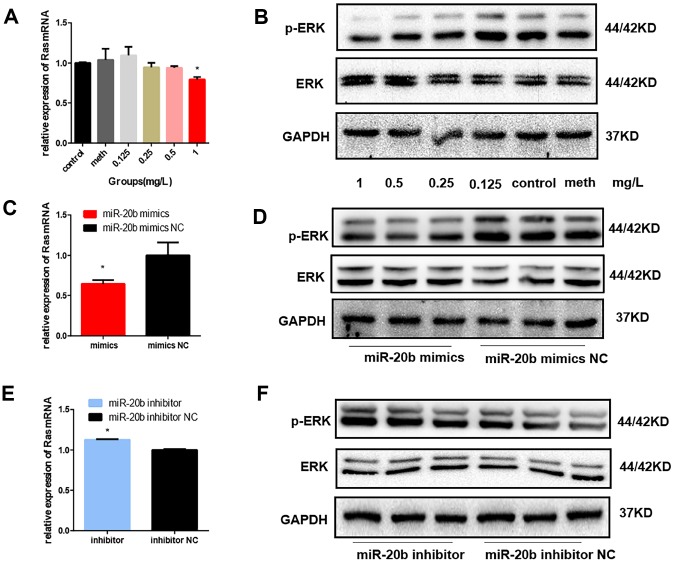
**Regulation of MAPK signaling pathway by PCB_1254_ and miR-20b.** (**A**) Total RNA was isolated from cells 24 hours after exposure of PCB1254, and the RNA level of Ras was gradually reduced. (**B**) Whole cell lysates were prepared 48 hours after exposure to PCB1254, and the results showed that as the concentration increased, the level of p-ERK1/2 gradually decreased. (**C**–**F**) the expression of Ras was decreased and the phosphorylation level was gradually decreased in the miR-20b overexpression group, whereas the silencing group was the opposite. (*: P<0.05).

FGF2 has been reported to be associated with photoreceptor cell proliferation by activating the downstream GRB2-RAS-MAPK pathway. The mRNA level of the Ras gene was detected by RT-qPCR, and total ERK1/2 and phosphorylated ERK1/2 (p-ERK1/2) protein expression levels were also detected. In the PCB_1254_ exposure group and the miR-20b overexpression group, we found that Ras mRNA levels were decreased (Figure 8A, 8C, 8E), and p-ERK1/2 protein levels were significantly down-regulated, while no significant differences was observed in t-ERK1/2 protein levels (Figure 8B, 8D, 8F). The results showed that PCB_1254_/miR-20b inhibited photoreceptor cell proliferation through MAPK/ERK pathway.

### The zebrafish model confirmed the inhibitory effects of miR-20b

**Figure 9:**
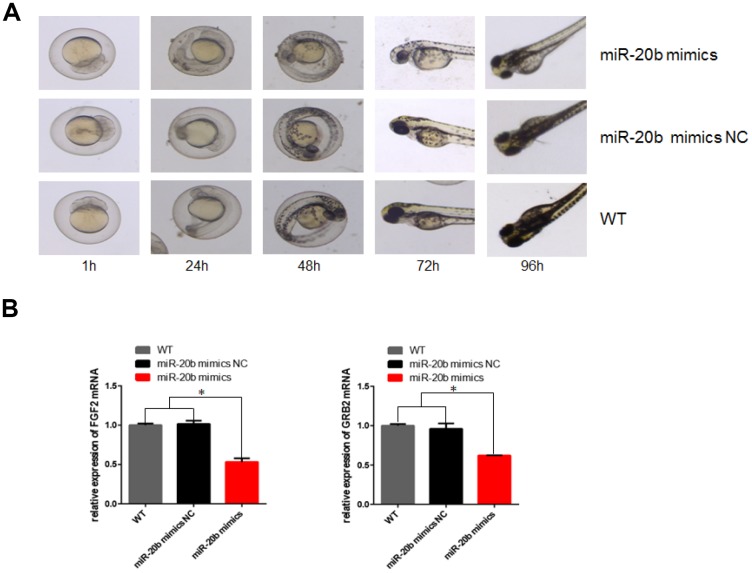
**Effect of mir-20b on zebrafish embryos.** (**A**) Injection of 4uM dre-miRNA-20b mimic and NC simulation into zebrafish embryos, embryo morphology at different time points. (**B**) Total RNA isolated from 96h post-injection embryos was used to determine FGF2 and GRB2 levels by real-time RT-PCR. The results showed that RNA expression was reduced compared to the control group.

**Figure 10:**
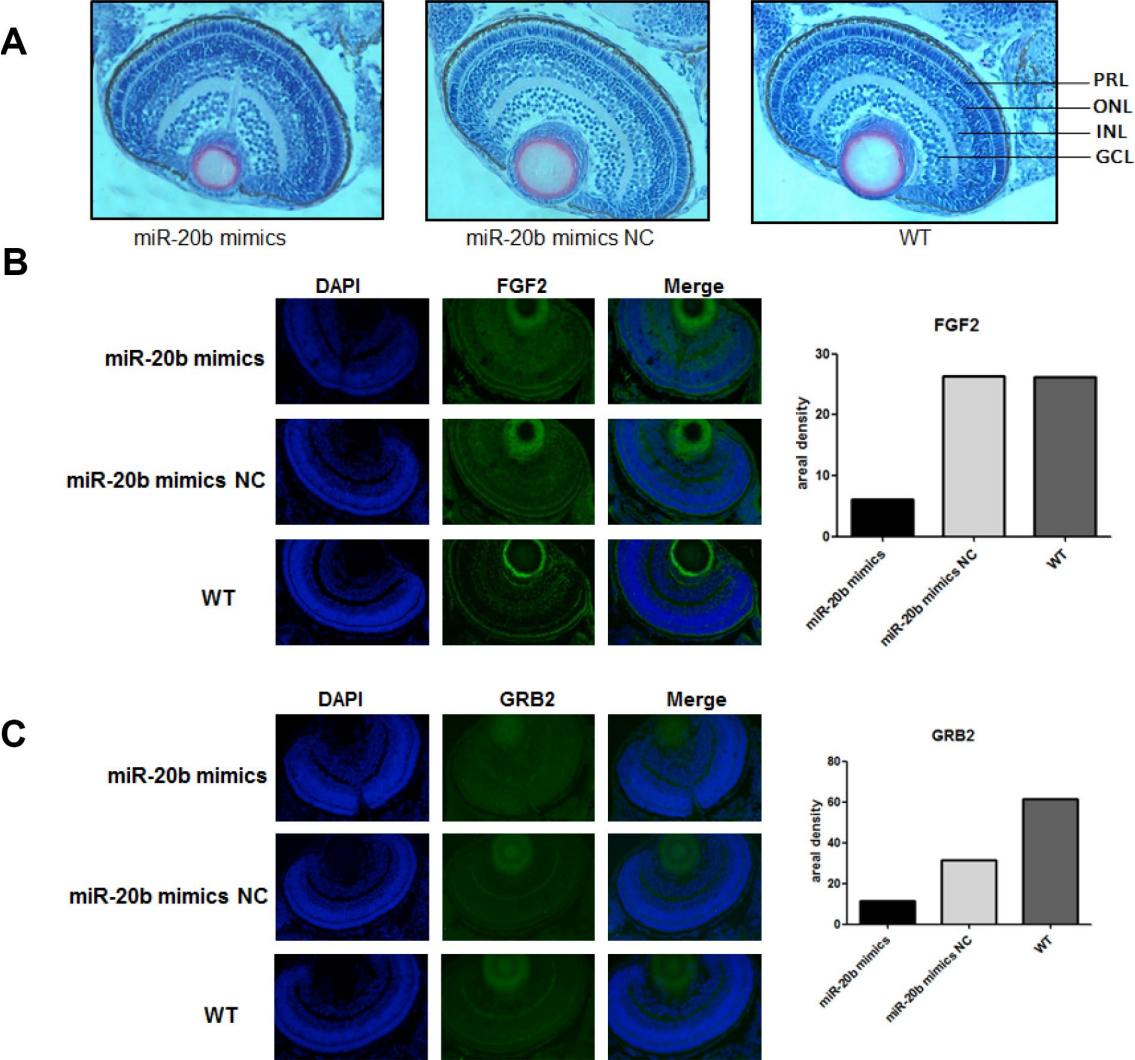
**The zebrafish model confirms the inhibitory effect of miR-20b.** (**A**) Observation of retinal tissue sections at 96hpf. We found that in the miRNA-20b mimic group, there were fewer cells in the photoreceptor layer than in the control and wild groups, and the arrangement of cells was disordered. PRL: Photoreceptor layer, ONL: Outer nuclear layer, INL: Inner nuclear layer, GCL: Ganglion cell layer. (**B**, **C**) Immunofluorescence detected decreased FGF2 and GRB2 brightness compared to controls. Relative fluorescence values were analyzed using Imagej software (*P<0.05).

Zebrafish model was used to observe the effects of miR-20b on embryonic and ocular development. There were more overall morphological abnormalities, especially the eye volume was significantly reduced in the 4 uM dre-miR-20b group (Figure 9A). In the 4 uM dre-miR-20b group, the embryo mortality rate was significantly higher and the survival rate was significantly reduced compared with the NC group and wild type group (Table 1). Under optical microscope, abnormal retinal morphology was found and characterized by sparse and irregular photoreceptor cells in the dre-miR-20b group. The thickness of the photoreceptor cell layer was significantly increased, while the number was obviously decreased compared with the NC group and the wild type group (Figure 10A). The immunofluorescence test results showed that compared with the NC group and wild type group, FGF2 and GRB2 were obviously expressed in the photoreceptor cell layer, while the expression levels of FGF2 and GRB2 protein in dre-miR-20b group were decreased (Figure 10B, 10C). Besides, the mRNA expressions of FGF2 and GRB2 in dre-miR-20b group were detected significantly lower than those in NC group and wild-type group (Figure 9B).

**Table 1:** Inhibition of zebrafish by miR-20b overexpression [n (survival rate,%)].

**Groups**	**Time(hpf)**	
	**72**	**96**
miR-20b mimics	38(54.3)*	35(50.0)*
NC	62(86.1)	61(84.7)
WT	76(97.4)	75(96.2)

*Compared with the normal control group P < 0.05.

## DISCUSSION

Nowadays, the incidences of amblyopia, refractive error and other non-infectious eye diseases are increasing. These eye diseases not only cause low binocular visual function in children, but also affect their quality of life and hinder their career development. Visual handicap can have a serious impact on the mental health of the children who suffer from it and can also bring heavy financial burden to the families and society. The number of people with visual disabilities in the world has reached 285 million and there are about 19 million children with visual impairment and about 500,000 new children with visual impairment each year.

Environmental factors have an increasingly severe impact on the visual health of children [16]. It has been reported that zebrafish exposed to higher concentrations of PCBs showed deformities during early developmental stages, such as yolk sac edema, pericardial edema, and reduced caudal curvature [17]. What’s more, PCB_1254_ has an adverse effect on the development of the zebrafish retina, primarily by affecting the retinal layer and the photoreceptors' differentiation, and at the same time, the continuous exposure to PCB_1254_ leads to abnormal OMR behavior in zebrafish larvae [7]. However, the molecular mechanism of PCBs-induced retinal dysplasia remains unclear.

In our study, we found that the expression of miR-20b was increased when exposed to PCB1254. MiR-20b is associated with the occurrence of cancer, sarcoma, inflammation, myasthenia gravis, premature senility, asthma and preeclampsia [18]. However, whether it contributes to the occurrence of retinal diseases such as amblyopia has not been reported yet. In this study, we found that miR-20b overexpression has a detrimental effect on retinal cells and we used prediction methods and subsequent experiments to confirm that miR-20b targeted both FGF2 and GRB2. FGF2, a member of the FGFs family, plays an important role in many cellular processes, including cell survival, proliferation, differentiation and migration, thus regulating a large number of developmental processes [19]. FGF2 was the first protein identified to affect photoreceptor cell differentiation. FGF2-like molecules may be secreted by neighboring cells, such as the retinal pigment epithelium, and they are involved in retinal development and differentiation [20]. Recent studies have shown that FGF2 plays an important role in the treatment of neuroretinal degeneration, myopia, vitreous lesions and in the protection of photoreceptors from oxidative damage [21]. GRB2 is a cytoplasmic protein that contains the src-homology 3 (SH3) domain flanking two single SH2 domains and plays an important role in the coupling of receptor tyrosine kinases to downstream signaling [22]. We further found that the expression of FGF2 and GRB2 in the miR-20b overexpression group was reduced, which was consistent with the results of the PCB exposure group. To determine the functional relevance between miR-20b and the target genes, we constructed plasmids of the interference and overexpression sequences of FGF2 and GRB2, respectively. Our results showed that miR-20b inhibited cell proliferation and promoted cell apoptosis via directly targeting FGF2 and GRB2.

It has been confirmed that FGF2, GRB2 were Mitogen-activated protein kinase (MAPK)MAPK signaling pathway upstream activator [23]. MAPK signaling pathway is essential for many cellular functions, including a variety of important physiological / pathological processes that regulate cell growth, differentiation, stress, and inflammation [24]. MAPK signaling pathway includes three classic pathways ERK, JNK and P38. Extracellular signal-regulated kinases (ERKs) 1 and 2 are the most well-characterized MAPKs [25]. In the ERK pathway, the most important upstream activator is Ras. After Ras is activated, the serine / threonine kinases Raf and MEK are activated in turn, and finally ERK is activated, completing the tertiary activation pathway of Ras-Raf-MEK-ERK to participate in various pathological / physiological processes of cells [26]. Through this study, we found that Ras expression was decreased and MAPK phosphorylation pathway was inhibited in the PCB_1254_ exposed group and the miR-20b overexpression group.

As a highly visual species, zebrafish responds visually at 72 hours post fertilization (hpf), when the retina is anatomically and functionally similar to adult retina morphology. And the zebrafish's eyes overall have a larger size compared with other fish, which makes it easier to observe [27]. Like most extant vertebrates, the zebrafish retina consists of seven major cell types derived from neuroectoderm, six neurons and a single glial cell (Müller cell); three distinct layers of cells, namely Ganglion cell layer, inner nuclear layer, photoreceptor cell layer [28]. 70% of human genes have at least one zebrafish ortholog, and 84% of known human pathogens have zebrafish counterparts. Zebrafish therefore are proved to be the dominant model organism for understanding human diseases. Photoreceptor cells play a very important role in the structure of the retina. Zebrafish, like humans, have two types of photoreceptor cells, namely, rod and cone cells. Results from zebrafish embryo miR-20b injection experiments, RT-qPCR, HE staining and immunofluorescence all showed that the expression levels of FGF2, GRB2 were decreased, while the mortality rate was increased, and the hatching rate and the number of retinal cell layers were both decreased, leading to arranged disorder. This was also consistent with the effect of PCBs on zebrafish embryos. Thus, we think that PCBs could affect retinal cell development through miR-20b/ FGF2 / GRB2 signaling pathways, resulting in visual dysfunction.

In conclusion, this study showed that PCBs affected the development of retinal cells by targeting the miR-20b /FGF2 / GRB2 / ERK signaling pathways, resulting in visual dysfunction. The discovery of this molecular mechanism may provide a new perspective for the pathogenesis of visual impairment and miR-20b may be a potential new therapeutic target.

## MATERIALS AND METHODS

### Animal maintenance

Wild type Tübingen zebrafish (Danio rerio) was purchased from the Model Animal Center of Nanjing University, Nanjing, China. The zebrafish were kept in a circulating cycle at a temperature of 28 ± 0.5 °C for 14 hours of light / 10 hours of darkness and fed twice daily. The embryos were collected by natural mating and randomly assigned to a Petri dish containing Hank's balanced salt solution (0.10g NaCl, 0.03g KCl, 0.04g CaCl_2_, and 0.16g MgSO_4_ in 100 mL double-distilled H_2_O). Triazolenesulfonate (MS-222) (0.168mg/mL) was used as a zebrafish anesthetic in zebrafish embryos experiment. All animal breeding and experimental procedures are approved by the Nanjing Medical University Animal Protection and Use Committee, Nanjing, China.

### Cell culture and toxin exposure

661w cells (photoreceptor cell lines), the internationally accepted rat retinal photoreceptor cell line, were purchased from American Type Culture Collection (ATCC, USA). Cells were cultured in Dulbecco’s Modified Eagle’s Medium (DMEM; Invitrogen, USA) containing 10% fetal bovine serum (FBS; Gibco, USA) and 1% penicillin / streptomycin. All cells were incubated in a 37 ° C, 5% CO_2_ environment. Aroclor1254 was 98% pure, purchased from Sigma, USA, dissolved in methanol (analytically pure, China) at a concentration of 1g/L (Lot No. 48586) (size 50mg). According to the principle of toxicology, PCBs of 0.125, 0.25, 0.5 and 1mg/L were prepared 3 times respectively, and blank control group and 0.01% methanol control group were prepared to eliminate the interference of cosolvent.

### MicroRNA mimic injection and morphological observation

4 uM dre-miR-20b mimics and corresponding negative control (NC) were injected with microinjector at single cell stage and collected in culture medium at 28.5°C. The developmental changes of zebrafish embryos were observed at 2 h, 12 h, 24 h, 48 h, 72 and 96 h, and the number of hatchlings and number of zebrafish were recorded. And dead embryos were cleared timely. Embryos were collected at the end of the experiment at 96 h.

### MiRNA, target gene siRNA and target gene overexpression plasmid transfection

The mmu-miR-20b mimic, mimic NC, mmu-miR-20b inhibitor, inhibitor NC, FGF2 siRNA, GRB2 siRNA, FGF2-pEX-2, GRB2-pEX-2 were purchased from GenePharma. Transfection was performed using Lipofectamine 2000 reagent according to the manufacturer’s guidelines (Invitrogen). 50nM miRNA mimic, 50nM miRNA inhibitor, 50nM siRNA, 0.5ug/mL overexpression plasmid were the final transfection concentrations.

### Construction of double luciferase reporter plasmids

We predicted the region of the miR-20b-targeted mouse FGF2-3-′UTR and GRB2-3-′UTR by miRanda (http://www.microrna.org/microrna/home.do) and TargetScan (http://www. targetscan.org/). The direct targeting was verified by the synthesis of the psiCHECK-2 vector (Generay, Shanghai, China) containing the wild-type or mutant miR-20b binding site in FGF2-3-′UTR and GRB2-3-′UTR. HEK293T cells were used as tool cells and were seeded into 6-well plates one day before transfection and then co-transfected with 1.6ug of target reporter plasmid and 50nM miR-20b mimic, NC, miR-20b inhibitor or inhibitor NC using Lipofectamine TM 2000 (Invitrogen). After 48-72 hours, luciferase activity was measured using a double luciferase kit (Promega, Madison, USA).

### Real-time quantitative polymerase chain reaction (RT-qPCR) assay

Total RNA was extracted with TRIzol reagent (Invitrogen Life Technologies, USA) from cells or tissues according to the manufacturer’s instructions. For miR-20b, miRNeasy Mini Kit (Qiagen Inc., Valencia, USA) was used for RNA reverse transcription. The mouse FGF2, GRB2, SWS1, CRX, Rho cDNA were reverse-transcribed using the commercial Revert Aid First Strand cDNA Synthesis Kit (Thermo Fisher Scientific, USA). Quantitative real-time RT-qPCR was performed with PowerUp SYBR Green Master Mix (Thermo Fisher Scientific, USA). U6 and GAPDH were used as internal controls for miR-20b and FGF2, GRB2, SWS1, CRX, Rho analysis, respectively. The RT-qPCR primers were designed by Generay (Shanghai, China). The reaction mixture was subjected to an initial step of 10 minutes at 95 °C followed by 40 cycles of 95 °C, 15 seconds, 60 °C, 1 minute, 72 °C, 45 seconds. The relative gene expression was analyzed by 2^-ΔΔCt^ method.

### Western blot analysis

Total protein was extracted using RIPA lysis buffer (Sigma) containing protease and phosphatase inhibitors (Beyotime, China) and quantified using the BCA kit (Beyotime, China). Proteins (30 mg of total protein) were separated by 12% or 10% SDS-PAGE and transferred to a polyvinylidene difluoride (PVDF) membrane (Millipore, USA). The membranes were blocked in TBST containing 5% nonfat milk, and incubated overnight at 4°C with various primary antibodies, including anti-FGF2 antibody (Absin); anti-GRB2 antibody (Abcam); anti-ERK1/2 antibody (CST); anti-pERK1/2 antibody (CST); anti-Ras antibody(Abcam); anti-β-Actin antibody(CST); anti-GAPDH antibody(Abcam). Next, it was incubated with an HRP-conjugated goat anti-rabbit IgG (CST). The values of band intensities were detected by ECL kit (Beyotime, China).

### Hematoxylin-eosin staining and immunofluorescence

Wild larvae and larvae injected with dre-miRNA-20b mimics, NC were sacrificed 96h after incubation. Approximately 60 larvae were used for light microscope analysis of the retinal layer. Embryos were anesthetized with 3-aminobenzoate and fixed with 4% paraformaldehyde. Lambs were washed in 0.01M phosphate buffered saline (pH 7.2), dehydrated in varying concentrations of ethanol, permeated and embedded in paraffin blocks. Each block was continuously transected at 2μm. The slices were hydrated, stained with hematoxylin and eosin, dried, and loaded in neutral balsam. Retinal layer thickness and 96hpf cell density were evaluated for each of six larvae in each group. Immunofluorescence staining was performed by hydration of the sections and microwave-assisted thermal repair of the antigen, followed by blocking with a 5% BSA blocking buffer in a humid chamber at 37°C for 30 minutes. And then primary antibody was added for incubation (4°C) overnight before dropwise addition of fluorescently labeled dimples for 1 hour, and lastly dropwise addition of DAPI for 2 minutes in the dark. Results were observed under a fluorescence microscope and recorded.

### Cell function assay

Exposed or transfected 661w cells were seeded in 96-well plates (1 × 10^3^ cells / well). At 0h, 24h, 48h and 72h, 10μL Cellell® Counting Kit-8 (CCK-8) solution (Dojindo Laboratories) was added to each well for 1h. The absorbance was measured at 450nm using a microplate reader (Bio-Rad) to detect proliferation. Cells were harvested 72 hours after exposure or transfection, fixed in 75% ethanol, overnight at 4 ° C, and then treated with 20 μl of RNase A at 37 ° C and 400 μl of propidium iodide at 4 ° C for 30 minutes. The cell cycle was detected using BD Accuri C6 flow cytometer (BD Biosciences) and FlowJo 7.6 software analysis. We used two methods to detect apoptosis. One was to collect the cells with trypsin/EDTA (Gibco BRL, USA) and wash them with phosphate-buffered saline (PBS, Gibco BRL). The cells were centrifuged and resuspended in 1mL of buffer and incubated with 5 mU/L of PE and 5 mU/L of 7-AAD for 15 minutes at room temperature (Biovision, CA, USA). The cells were then analyzed immediately using flow cytometry. Analysis of apoptotic cells was carried out using the Caspase-3 spectrophotometric kit (KeyGEN BioTECH, China). Absorbance values were measured on a microplate reader at 400-405 nm to detect cell apoptotic. The experiments were repeated three times.

### Statistical analysis

The experimental data used SPSS19.0 software to establish a database, with mean ± standard deviation (SD). The experimental data of each group were tested by homogeneity of variance, and the data between groups were compared using one-way ANOVA and pairwise comparison. P <0.05 for the difference was considered statistically significant.
